# Using Community Ecology Theory and Computational Microbiome Methods To Study Human Milk as a Biological System

**DOI:** 10.1128/msystems.01132-21

**Published:** 2022-02-01

**Authors:** Liat Shenhav, Meghan B. Azad

**Affiliations:** a Center for Studies in Physics and Biology, The Rockefeller University, New York, New York, USA; b Department of Pediatrics and Child Health, University of Manitoba, Winnipeg, Canada; c Manitoba Interdisciplinary Lactation Centre, Children’s Hospital Research Institute of Manitoba, Winnipeg, Canada; University of California San Diego

**Keywords:** computational methods, human microbiome, human milk, chronobiology, community ecology theory, system biology, lactation, breastfeeding

## Abstract

Human milk is a complex and dynamic biological system that has evolved to optimally nourish and protect human infants. Yet, according to a recent priority-setting review, “our current understanding of human milk composition and its individual components and their functions fails to fully recognize the importance of the chronobiology and systems biology of human milk in the context of milk synthesis, optimal timing and duration of feeding, and period of lactation” (P. Christian et al., Am J Clin Nutr 113:1063–1072, 2021, https://doi.org/10.1093/ajcn/nqab075). We attribute this critical knowledge gap to three major reasons as follows. (i) Studies have typically examined each subsystem of the mother-milk-infant “triad” in isolation and often focus on a single element or component (e.g., maternal lactation physiology or milk microbiome or milk oligosaccharides or infant microbiome or infant gut physiology). This undermines our ability to develop comprehensive representations of the interactions between these elements and study their response to external perturbations. (ii) Multiomics studies are often cross-sectional, presenting a snapshot of milk composition, largely ignoring the temporal variability during lactation. The lack of temporal resolution precludes the characterization and inference of robust interactions between the dynamic subsystems of the triad. (iii) We lack computational methods to represent and decipher the complex ecosystem of the mother-milk-infant triad and its environment. In this review, we advocate for longitudinal multiomics data collection and demonstrate how incorporating knowledge gleaned from microbial community ecology and computational methods developed for microbiome research can serve as an anchor to advance the study of human milk and its many components as a “system within a system.”

## STUDYING HUMAN MILK AS A “SYSTEM WITHIN A SYSTEM”: RATIONALE, CHALLENGES, AND A NOVEL STRATEGY

Human milk is a dynamic and responsive fluid that is uniquely suited to the infant’s nutritional and immunological needs, providing the foundation for healthy growth and development (1, 2). Lactation has evolved over millions of years to support the survival of mammalian offspring and their developing microbiomes (3). Besides essential nutrients, human milk is rich in cytokines, immunoglobulins, growth factors, soluble receptors, immune cells, enzymes, oligosaccharides, and microbiota (4–6). These components are dynamic and change over time as the infant’s needs and maternal physiology evolve across lactation, with some components also having diurnal rhythms and/or fluctuations during a single feeding.

It is well established that breastfeeding is protective against infections and may also help prevent immune-mediated diseases, both during and beyond the lactation period (7, 8). For example, breastfed infants have a lower incidence of diarrhea, a lower risk of mortality due to infectious disease, and a reduced risk of childhood asthma (8). While these associations are complex and not solely attributable to the composition of human milk (the act of breastfeeding likely contributes and confounding is also possible), it is clear that human milk has unique immunomodulatory properties that are not replicated in commercial infant formulas (9). Yet, despite its extraordinary importance, much remains unknown about the ecology of human milk, its contribution to normal development, and its influence on infant and maternal health. We attribute this knowledge gap, at least in part, to the undercharacterized interplay among different milk components and between milk, mother, and infant, referred to as a “triad” by Bode et al. (10).

**FIG 1:**
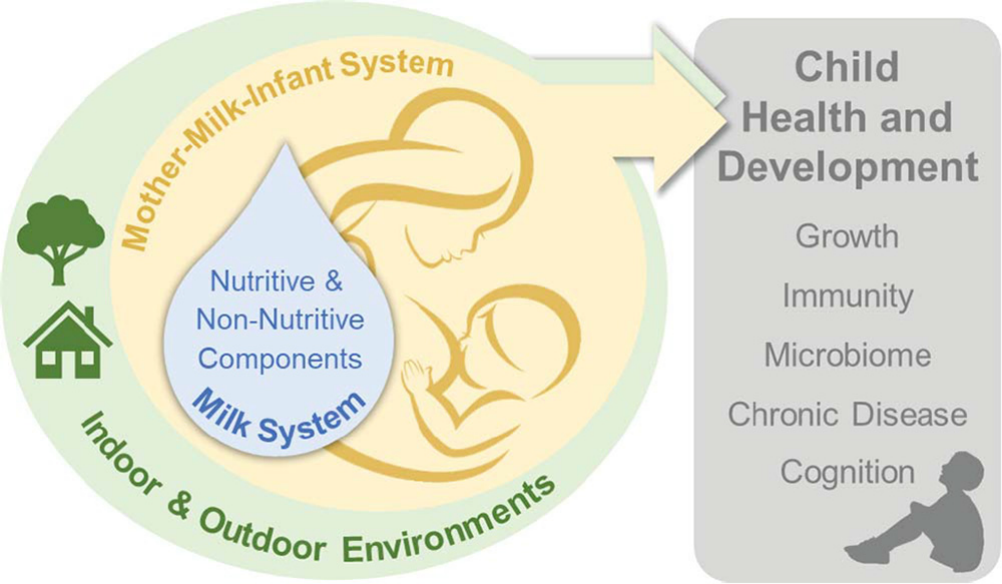
The mother-milk-infant triad and its environment as the unit of study.

This observation was recently reinforced by the U.S. National Institutes of Health and the Bill and Melinda Gates Foundation, advocating for a much-needed shift in the conceptual approach to studying human milk as a “system within a system” (11). The common, yet overly simplistic approach to analyzing single, mostly nutritive components of human milk was deemed inadequate in many cases, as it underestimates the importance of the chronobiology and systems biology of human milk. Of note, the biological impact of human milk depends not only on its composition but also on how it is fed and the physiology of the recipient infant. For example, the expression of specific receptors on the nasal and gut epithelium will mediate the response to various human milk components. Epithelial permeability will also determine the ability of human milk macromolecules to be absorbed into circulation. These examples emphasize the need to study human milk as a system in the context of the “mother-milk-infant triad” (10) and environmental factors affecting it (Fig. 1).

From a data science perspective, to comprehensively study complex biological processes such as human milk and lactation, it is essential to take an integrative and “multilayer” approach that accounts for the interactions among individual components and their collective functions. For human milk, this includes multiple nutrients and nonnutritive components that can be profiled with targeted assays or untargeted omics approaches (e.g., proteome, microbiome, metabolome, etc.). However, it is computationally challenging to integrate signals from multiple modalities—especially temporal ones—in a unified model. In this review, we propose a novel strategy to mitigate these challenges by leveraging computational methods developed to study another complex system, the human microbiome. Specifically, we suggest defining and studying human milk as a community or an ecosystem, inspired by microbial communities, by using concepts such as succussion, diversity, and keystone components. We further suggest leveraging microbiome methods and community ecology theory to develop computational approaches to study human milk as a system.

**FIG 2:**
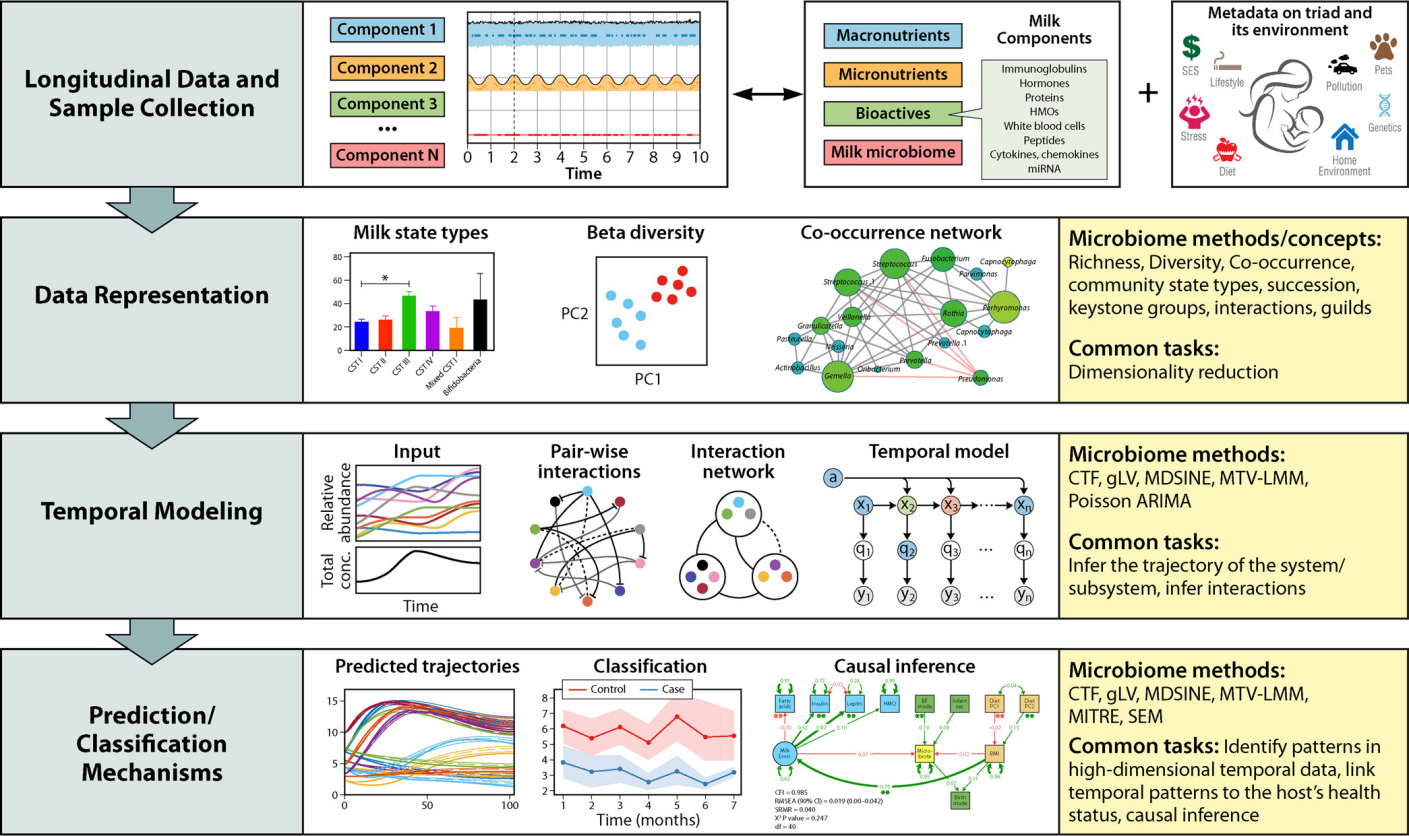
Methodology for incorporating microbial community ecology and computational microbiome methods in the study of human milk. Longitudinal data collection involves collecting dense (frequent) human milk samples from women in tandem with high-dimensional metadata that capture the context (e.g., health, diet, environment) of the mother-milk-infant triad together with microbiome samples from both the mother and infant. Data representation entails applying computational methods or ecological concepts that summarize high-dimensional data, extracting important underlying structures in the data, and linking them to clinical outcomes. These concepts/methods include community state types, richness, diversity, co-occurrence networks, interactions, and more. Temporal modeling is inferring dynamical systems from milk time series data, using 4 steps as follows: (i) input is a time series of abundances of actors in the system or some lower-dimensional representation of the system over time (e.g., diversity over time); (ii) pairwise interaction network reflecting nonzero interaction coefficients in underlying dynamical systems model; (iii) interaction network with interaction module structure; and (iv) temporal model unrolled in time to explicitly show temporal dependencies. This schematic is inspired by methods developed for microbial dynamics (57). In predict/classify/elucidate mechanisms, by using data summaries extracted from the data representation methods/concepts as well as the temporal modeling, we can characterize the dynamics of human milk components, predict infant outcomes, and elucidate mechanisms underlying them. This can be done using statistical/probabilistic models, machine learning algorithms, mechanistic models that rely on ecology theory, and causal inference. Relevant microbiome methods include compositional tensor factorization (CTF) (63), generalized Lotka-Volterra (gLV) (57), Microbial Dynamical Systems INference Engine (MDSINE) (77), microbial temporal variability linear mixed model (MTV-LMM) (59), Poisson ARIMA (58), Microbiome Interpretable Temporal Rule Engine (MITRE) (64), and structural equation modeling (SEM) (78, 79).

We suggest that modeling the human microbiome shares many conceptual similarities with the modeling of human milk, including its microbial and nonmicrobial components. Conceptually, both the microbiome and human milk are dynamic ecosystems, requiring ecological measurements (e.g., diversity) and computational methods that can identify patterns from high-dimensional data and link these patterns to the host’s status or development. Moreover, in both cases, such computational methods must contend with numerous challenges shared among the two systems, including measurement noise, sparse and irregular temporal sampling, and intersubject variability. Finally, we note that although the omics era has accelerated all aspects of biological research, its effects have been particularly apparent in studies of microbial communities and the human microbiome (12–17). Thus, computational frameworks developed to study the microbiome are particularly well suited for adaptation to fit the unique characteristics of human milk research (18). In this review, we showcase the wide array of parallels between human milk and the microbiome and demonstrate how incorporating knowledge gleaned from microbial community ecology and computational microbiology can serve as an anchor to advance the study of human milk as a system. We advocate for the collection of dense longitudinal data and development of tailored computational methods to represent this system and elucidate its emergent properties (Fig. 2).

## HUMAN MILK IS A COMPLEX ADAPTIVE SYSTEM. SO IS THE MICROBIOME

Ecosystems, such as microbial communities, are complex adaptive systems; they are complex because they have many parts and many connections between the parts, and they are adaptive because their feedback structure gives them the ability to change in ways that promote survival of the ecosystem in a fluctuating environment. These are dynamic systems able to *adapt in* and *evolve with* a changing environment. The key to understanding these ecosystems lies in emergent properties: the distinctive features and behaviors that “emerge” from the way that complex adaptive systems are organized. Emergent properties are usually defined as the output resulting from an interacting set of variables within a system. For example, at the organismal level, vision and color perception are emergent properties that result from the interaction of different chemical signals between different cell types in the eye and brain. Just as vision cannot be understood by studying a single cell type or a single signaling molecule, the emergent properties of human milk cannot be understood by studying a single milk nutrient or bioactive compound.

It was recently suggested that the infant gut microbiome is a complex adaptive system, crucial to the maintenance of various emergent properties such as infant immune system training (19). This emergent property is not attributable to a single component of the ecosystem; instead, it relies on a temporally structured pattern of bacterial diversity increase after birth and the succession of keystone groups of microbes. Similar to the gut microbiome, human milk supports a set of emergent properties contributing to the development of the nursing infant, including microbial dispersal and selection, physical growth, neurodevelopment, and immune system maturation (20–27). We thus suggest that human milk can also be considered a complex adaptive system in which both low-level local interactions and selection mechanisms combine to create high-level patterns. Notably, in the unique case of human milk, these properties originate in the mother but emerge in the infant, emphasizing the importance of the mother-milk-infant triad and its environment as the unit of study (10).

The emergent properties of a complex adaptive ecosystem are supported by combinations of diversity as well as keystone groups, both of which ensure community resilience and make it difficult to attribute a cause-effect relationship to individual features or groups (28). Studies of microbial communities have used multiple properties to characterize ecosystems, including species richness, diversity and functional profile, the level of interactions between species in the ecosystem, and the strength of these interactions (29). In ecology, these concepts are a way to thoughtfully summarize the ecosystem and describe it as a whole. In microbiome science, the use of community ecology theory mirrors the improvement in our understanding of these complex microbial systems, shifting from a singular to a multilevel perspective. We thus suggest applying community ecology theory to study human milk.

To achieve this, we can define milk as an ecosystem and borrow concepts from community ecology theory to characterize it. This will require defining the “actors” of this ecosystem (e.g., maternal cells, genes, nutrients, enzymes, immunoglobulins, microbial genomes), quantifying their levels, and describing community succession, richness, diversity, functional profile, keystone actors, and more. Studies in complexity theory (30, 31) suggest that these measurements, along with productivity, resilience, and biomass, are a comprehensive description of complex systems. These studies use mathematical models to examine how a large collection of components, locally interacting with each other at small scales, can spontaneously self-organize to exhibit nontrivial global structures at larger scales. Thus, to model human milk as a complex adaptive system, we suggest using the following concepts, which have been successfully applied in microbiome science and linked to clinical outcomes (see Table 1).

**TABLE 1:** Concepts from microbiome science and microbial ecology that can be applied to understanding the compositional and functional characteristics of human milk

Concept	Definition	Example applications in human microbiome science	Suggested applications in human milk science
Richness	The number of actors in a community.	Richness of the human gut microbiome (the number of different bacterial species) was associated with metabolic markers (80).	Can be used to represent the number of actors across all milk components or the number of actors within each subsystem (nutrients, bioactives, microbiome).
Diversity	Taxonomic diversity refers to the number and relative abundance of actors in a community. Functional diversity refers to the variety of processes or functions in a community that are important to its structure and dynamic stability.	Low diversity in the adult gut microbiome has been associated with acute diarrheal disease (81), inflammatory bowel disease (82), Clostridium difficile infection (83), liver disease, and in cancer patients (84).	Can be used to quantify the variability/similarity across milk components. Can be used as an aggregative measurement or within a subsystem.
Co-occurrence networks	A graphic visualization of potential relationships among actors in a system.	The patterns of species and strain co-occurrence in the vaginal microbiome were associated with adverse pregnancy outcomes (85).	Can be expanded to represent connections among a wide array of components (e.g., HMOs, nutrients, and microbes).
Community/system state types	A group of community states, where each state is characterized by similar composition and abundance.	In the vaginal microbiome, five CSTs were identified. These CSTs were associated with health outcomes such as preterm birth and bacterial vaginosis (36, 37).	Can be used to characterize state types using a single component like HMOs or characterize “overall milk state type” across all components (e.g., lactotypes [39]).
Keystone groups	Exceptionally important actors whose presence is crucial in maintaining the organization and diversity of the ecological system.	*Bifidobacterium* species and subspecies were suggested as keystone species in the infant gut, as they are well adapted to its transmission routes and growth conditions (19).	May help identify key milk components associated with predisposition to (or protection from) adverse infant or maternal outcomes (e.g., necrotizing enterocolitis, mastitis).
Interactions	The types and strengths of the relationships between actors in the system.	Microbial interactions in oral communities mediate biofilm properties and are associated with oral health and disease (86, 87).	May help establish a reference or baseline for milk interactions (rather than specific components) that are associated with optimal health outcomes (e.g., certain nutrients may be absorbed better in combination; immunoglobulins may interact with nonhuman antigens).
Guilds	Members are defined as belonging to the same guild if they exploit the same class of resources in a similar way or work together as a coherent functional group.	In microbiome science, a guild was defined as a group of bacteria that show consistent coabundant behavior and are likely to work together to contribute to the same ecological function (e.g., fermentation of indigestible dietary components) (76).	Could help identify groups of human milk components that show consistent coabundant behavior and are likely to work together to contribute to the same ecological function (e.g., immune training or gut barrier integrity).
Resilience	A system or community’s capacity to promptly return to its initial state after a perturbation.	This is a key characteristic of a healthy infant gut microbiome, protecting it from reaching a dysbiotic state (e.g., after antibiotic exposure) (88).	Defining human milk resilience will enable the development of effective interventions aimed at maintaining the emerging properties of human milk.
Succession	A pattern of temporal changes in specific composition after a radical disturbance or after the opening of a new niche in the physical environment for colonization.	Succession in the infant gut starts with the arrival of pioneer species that transform the gut habitat and enable the settlement of first succession species. This temporally structured process contributes to the identity and dynamics of the infant gut microbiome and thus plays a key role in immune development (53, 54).	Can be applied to determine the pioneer components in human milk (microbial species, immune factors, oligosaccharides, etc.) and understand: What influences their identity? What is the temporal structure of this community succession? What are the effects of different initial conditions (i.e., different pioneer components) on the composition and dynamics of other milk components?
Emergent property	A property which a complex system has but which its individual components do not have.	Immune development is an emergent property of the infant gut microbiome. This emergent property is not attributable to a single component of the ecosystem; instead, it seems to rely on a temporally structured pattern of bacterial diversity increase after birth and the succession of keystone groups of microbes (19).	Human milk supports a set of emergent properties contributing to the development of the nursing infant, including microbial dispersal and selection, physical growth, neurodevelopment, and immune system maturation.

### Richness.

Richness (32) is the number of actors in a community. In human milk, richness can be used to represent the number of actors, across all components, or the number of actors within each subsystem (e.g., HMOs, microbiota, immune factors).

### Diversity.

We define two types of diversity (33). First, there is taxonomic diversity, which refers to the number and the relative abundance of actors in a community. Second, there is functional diversity, defined as the variety of processes or functions in a community that are important to its structure and dynamic stability (e.g., pre- and probiotic properties, ability to support epithelial development, capacity to neutralize pathogens). Similar to richness, diversity in human milk can be an aggregative measurement across all components or within each component. We also suggest adapting the alpha- and beta-diversity measurements, representing compositional or functional diversity of a community and the similarity or dissimilarity across two communities, respectively (34).

### Co-occurrence networks.

Microbial co-occurrence networks (35) are widely applied to explore connections in microbial communities. Nodes and edges in microbial co-occurrence networks usually represent microbes and statistically significant associations between nodes, respectively. In human milk, co-occurrence networks can be expanded to represent connections among a wide array of components (e.g., human milk oligosaccharides [HMOs], nutrients, and microbes).

### Community state types.

The term “community state type” (CST) is used in microbial ecology to describe a group of microbial communities with similar composition and abundance (36, 37). In human milk, we can characterize state types using a single component like HMOs or characterize “overall milk state type” across all components. For example, Masi et al. showed that community state types of HMOs differentiate preterm infants that develop necrotizing enterocolitis from healthy ones (38). Another example is the concept of “lactotypes” (39). Munblit et al. suggested that women can be characterized according to their milk composition profile and that variation in combinations of milk components rather than single factors may be linked with infant health.

### Keystone groups.

Keystone groups (40) are exceptionally important actors whose presence is crucial in maintaining the organization and diversity of the ecological community. This concept can be applied to investigate whether there are equivalent “keystone” components or groups in human milk on which other actors in the ecosystem depend, such that if they were removed, the ecosystem would change drastically. For example, this view may help in identifying key milk components associated with adverse infant or maternal outcomes such as necrotizing enterocolitis or mastitis, respectively.

### Interactions.

Microbial interactions are crucial for a successful establishment and maintenance of a microbial population. In microbial communities, we know that species interact with one another in multiple ways. Positive microbial interactions include mutualism (cooperation), protocooperation (both benefit; however, both populations can survive on their own), and commensalism (one benefits, and the other is unaffected). Negative microbial interactions include predation, parasitism (one benefits; one is harmed), amensalism (one is harmed; the other is unaffected), and competition. We suggest that the type and strength of interactions between milk components may also play an important role and should therefore be characterized. This type of analysis may help us determine how human milk components relate to one another. We can use this approach to establish a reference or a baseline for milk interactions that are associated with optimal health outcomes (similar to establishing reference values of nutrients). We can also establish whether there is a conserved set of interactions between milk components or whether these interactions are context specific. For example, a recent study (41) showed that particular interactions between HMOs, milk microbiota, and the infant gut microbiome prevented or ameliorated neonatal rotavirus infections—a clinically important discovery that would not be possible by focusing on any component in isolation. Of note, different types of interactions must be considered and modeled to account for the different ways that living (e.g., maternal and microbial cells) and nonliving (e.g., nutrients, enzymes, HMOs) human milk components interact.

### Guilds.

Guilds (adapted from macroecology) (42) are an alternative to thinking about species and traditional taxonomy. Members are defined as belonging to the same guild if they exploit the same class of resources in a similar way or work together as a coherent functional group. In human milk, a guild can be a group of components that show consistent coabundant behavior and are likely to work together to contribute to the same function/emergent property, such as immune training or gut barrier function.

### Resilience.

Resilience (43) is a system’s or community’s capacity to promptly return to its initial state after a perturbation. Resilience is a crucial property of complex adaptive systems (44). In human milk, a perturbation can manifest as a change in maternal (antibiotics, illness, diet) or infant (e.g., antibiotics, infection, vaccine) factors. This would require a prospective longitudinal characterization of human milk before and after a disturbance (e.g., maternal/infant illness, antibiotics) to establish a return to initial state. We note that the identification of the critical events and factors that influence human milk resilience and function will enable the development of effective interventions aimed at maintaining the emerging properties of human milk (e.g., immune system development and disease prevention).

One example of an initiative that will enable such representation of human milk as an ecosystem is the International Milk Composition (IMiC) Consortium (https://www.milcresearch.com/imic.html). Using a comprehensive multiomics approach, the interdisciplinary IMiC team is studying 1,000 mother-milk-infant triads across diverse settings with the overarching objective to identify, comprehensively, human milk components linked to infant growth and resilience to inform maternal and infant nutrition recommendations and interventions. Another rare example of a multilayer human milk research effort that could apply our suggested approach is the INSPIRE project, where a vast array of milk components was analyzed from 400 women in 8 countries (45).

It is important to note that the ecological concepts described above, and both the IMiC and INSPIRE projects, capture only a “snapshot” of this dynamic system. New longitudinal studies are required to model the chronobiology and temporal changes in human milk composition occurring during lactation.

## HUMAN MILK IS A DYNAMIC SYSTEM. SO IS THE MICROBIOME

Just as microbial communities are dynamic ecosystems that change in response to internal interactions and external perturbations, human milk composition changes over the time course of lactation due to a combination of intrinsic and extrinsic factors (46, 47). By the end of pregnancy, the mammary gland starts to produce colostrum, which is especially rich in bioactive factors that provide passive immunity to the infant (2, 48). Transitional milk is secreted for about 2 weeks, followed by mature milk, which comprises a thinner “fore-milk” that becomes fattier toward the end of the feed, referred to as “hind-milk” (2). This temporally structured process suggests there is a relatively small set of conserved trajectories through which human milk changes during lactation, with some variation depending on individual circumstances. However, the granular temporal dynamics, or “chronobiology,” of human milk remain poorly characterized.

One temporally structured process that might share similarities with the chronobiology of human milk is microbial succession. In ecology, succession is defined as the pattern of changes in a community after a disturbance or after the opening of a new niche to colonization (49). Succession in the infant gut microbiome starts with the arrival of pioneer species that transform the gut habitat and enable the settlement of first succession species. This process is influenced by maternal factors (e.g., body weight and stress) (46, 50, 51), delivery mode (52–54), and infant diet (55, 56). The temporal structure of these environmental factors contributes to the identity and dynamics of the infant gut microbiome and thus plays a key role in infant development. We can use this knowledge as an inspiration to study the succession of all kinds of milk components: What are the pioneer components (microbial species, immune factors, oligosaccharides, etc.) in human milk? What influences their identity? What is the temporal structure of this community succession? What are the effects of different initial conditions (i.e., different pioneer components) on the composition and dynamics of other milk components?

Investigating the temporal dynamics of microbial communities has required development of specialized computational time series analysis tools. These methods, designed to extract rich information from longitudinal data, can be adapted to model the dynamics of human milk. For example, a common task in the analysis of longitudinal microbiome data is to infer the trajectories of the community as well as predict future compositions. This task is equally relevant to human milk. Both microbiome and milk longitudinal data may include many different temporal patterns such as cyclical effects (e.g., seasonal or circadian effects), long-term trends, or even delayed effects of shifts in composition. However, models may differ substantially in the types of temporal patterns they can infer and predict. For example, the generalized Lotka-Volterra model (57) is limited to capturing single time-step interactions (changes between two consecutive time points). Other models, such as the Poisson ARIMA, allow for temporal interactions to be carried over multiple time points (58). Even greater flexibility is achieved by models such as those of Shenhav et al. (59) and Silverman et al. (60), which allow for more complex time series modeling such as the inclusion of seasonal or polynomial trends. Other methods can achieve even greater flexibility by using nonparametric kernel methods (61) or finding low-dimensional representations of trajectories (62). In summary, multiple methods were developed in microbiome science to infer the trajectories of microbial communities (Fig. 2). These methods can be adapted to model human milk trajectories, with the added value of highlighting different temporal patterns and assumptions.

Another common goal is to simultaneously identify patterns from high-dimensional temporal data and link these patterns to the host’s (mother/infant) health status or development. For example, Martino and Shenhav et al. developed a dimensionality reduction method called compositional tensor factorization (CTF) (63) that decomposes a tensor (i.e., three-dimensional matrix) and relates microbial sequences, hosts, and time. Applying CTF to longitudinal infant gut data revealed a consistent microbial signature that differentiated infants by birth mode across the first years of life. Notably, this temporal separation between birth modes was significantly stronger than the separation observed using traditional, nontemporal methods. This model can be easily adapted to identify temporal milk trajectories by changing its input (from microbial abundance to milk composition). Properly adapted to human milk, CTF can potentially highlight temporal patterns leading to different infant or maternal phenotypes. Further, Bogart et al. (64) developed a supervised machine learning method for microbiome time series analysis that infers interpretable rules linking changes in abundance of clades of microbes over time windows to host phenotypes, such as the presence/absence of disease. This algorithm enables the discovery of biologically interpretable relationships between the temporal dynamics of the microbiome and the host. This method can also be adjusted to link human milk dynamics and infant or maternal health outcomes. Other relevant computational time series analysis tools in the microbiome include prediction of future behaviors of the ecosystem (65–67), inference of stability and response times to perturbations (67, 68), and discovery of causal interactions (69).

A straightforward approach common in microbiome studies that can be easily adapted in human milk is to identify important interactions between taxonomic/functional groups as well as using statistical covariation over time. Applying this approach to longitudinal gut microbiome data from healthy infants in a Bangladeshi birth cohort identified a network composed of 15 covarying bacterial taxa (70, 71). These “normal” microbial profiles enabled identification of (i) impaired microbiome development in other children with malnutrition, and (ii) complementary foods contributing to healthy microbiome restoration (70, 71). In the context of human milk, this approach can be used to define normal milk profiles across multiple milk components.

To enable such analysis of human milk, dense, comprehensive, longitudinal data should be collected. A seminal example of such a study in the microbiome space is the Environmental Determinants of Diabetes in the Young (TEDDY) cohort (72). This study densely sampled and modeled the temporal development of the gut microbiome among 900 infants, which enabled the discovery and characterization of the structural and functional assembly of the microbiome in early life. In human milk, the Mothers, Infants and Lactation Quality (MILQ) study is a relevant longitudinal study of 1,000 mothers aimed to establish reference values for human milk nutrients and bioactives analyzed at 4 time points in the first 9 months of lactation (73). Of note, most of the statistical methods described above would require a denser sampling scheme (i.e., daily, weekly) to enable accurate inference and prediction.

## LOOKING FORWARD: DECIPHERING HUMAN MILK ECOLOGY

To address the methodological gaps of modeling milk as a system, we advocated, in this review, to use the microbiome as an inspiration and anchor. Specifically, we proposed to use the knowledge gleaned from microbial community ecology and computational microbiome methods as a starting point in modeling this complex system. To fully unlock the potential of this approach, it is essential to record, represent, and decipher the mother-milk-infant triad during lactation.

### Record: longitudinal data collection.

We need to collect dense, ideally daily, human milk samples from women across the globe in tandem with high-dimensional metadata that capture the context of the mother-milk-infant triad and its environment (e.g., demographics, genetics, lifestyle) and microbiome samples from the mother and infant (gut, vaginal, oral, nasal, skin). Similar to the IMiC project, the analysis of these samples should include macronutrients, micronutrients, oligosaccharides, growth factors, immunoglobulins, cytokines, metabolites, and microbes using assays validated and standardized for human milk. This will allow us to answer fundamental questions, such as: What is the variation in milk composition over time? Why does it change? How does it change (jumping between discrete states? continued change? Does one component change more than others)?

### Represent: multilayer data representation.

Given that these multilayer data are high dimensional, we need to represent them in a meaningful way. One strategy is to assume that milk profiles that lead to optimal health outcomes harbor an underlying and low-dimensional structure that informs their dynamics and the interactions between their components. This low-rank representation of the data can be done either by (i) using machine learning dimensionality reduction or feature selection methods, and (ii) using complex adaptive systems and community ecology. Dimensionality reduction methods (e.g., principal-component analysis [PCA], t-stochastic neighbor embedding [t-SNE]) transform the data from a high-dimensional space into a low-dimensional space such that the latter retains some meaningful properties of the original data. Feature selection methods (e.g., LASSO) reduce the number of data features that are relevant to a specific prediction task. We thus postulate that both machine learning methods and ecology theory can be adapted and extended to study human milk as a system.

### Decipher: prediction, mechanistic modeling, and causal inference.

Finally, we wish to use this representation of the data to characterize the chronobiology of human milk, predict infant outcomes, and elucidate mechanisms underlying them. This can be done using statistical/probabilistic models, machine learning algorithms, mechanistic models that rely on ecology theory, and causal inference. We note that in order to build an integrated predictive model across different data modalities, multimodal analysis and ensemble techniques (e.g., stacked generalization) should be used (74, 75). As for mechanistic models that rely on community ecology theory, if we model human milk as a complex adaptive system, we can infer and predict its productivity, resilience, and the interactions between its functional groups. If we use the concept of guilds (76), we can identify candidate functional groups/components that may causatively contribute to infant health outcomes. With respect to chronobiology, we can extend mechanistic models such as the generalized Lotka-Volterra to infer dynamics and interactions between the functional groups/components of the ecosystem (57).

In summary, we suggest that similar to the human microbiome, human milk can be defined and modeled as a complex adaptive system. The dynamic nature and emergent properties of this ecosystem highlight the necessity of longitudinal multilayer human milk studies, along with tailored computational methods, which will eventually allow us to identify and characterize milk interactions and dynamics that are associated with optimal health outcomes. In this review, we advocate that knowledge gleaned from microbial community ecology and computational microbiology can serve as an anchor to advance the study of human milk and its many components as a complex adaptive “system within a system” that is vital to maternal and infant health.
